# Hybrid Deep Learning Predictor for Smart Agriculture Sensing Based on Empirical Mode Decomposition and Gated Recurrent Unit Group Model

**DOI:** 10.3390/s20051334

**Published:** 2020-02-29

**Authors:** Xue-Bo Jin, Nian-Xiang Yang, Xiao-Yi Wang, Yu-Ting Bai, Ting-Li Su, Jian-Lei Kong

**Affiliations:** 1 School of Computer and Information Engineering, Beijing Technology and Business University, Beijing 100048, China; yangnianxiang@st.btbu.edu.cn (N.-X.Y.); baiyuting@btbu.edu.cn (Y.-T.B.); sutingli@btbu.edu.cn (T.-L.S.); kongjianlei@btbu.edu.cn (J.-L.K.); 2 China Light Industry Key Laboratory of Industrial Internet and Big Data, Beijing Technology and Business University, Beijing 100048, China; 3 Beijing Key Laboratory of Big Data Technology for Food Safety, Beijing Technology and Business University, Beijing 100048, China

**Keywords:** sensing data prediction, EMD, convolution operation, GRU, smart sensing, IoT

## Abstract

Smart agricultural sensing has enabled great advantages in practical applications recently, making it one of the most important and valuable systems. For outdoor plantation farms, the prediction of climate data, such as temperature, wind speed, and humidity, enables the planning and control of agricultural production to improve the yield and quality of crops. However, it is not easy to accurately predict climate trends because the sensing data are complex, nonlinear, and contain multiple components. This study proposes a hybrid deep learning predictor, in which an empirical mode decomposition (EMD) method is used to decompose the climate data into fixed component groups with different frequency characteristics, then a gated recurrent unit (GRU) network is trained for each group as the sub-predictor, and finally the results from the GRU are added to obtain the prediction result. Experiments based on climate data from an agricultural Internet of Things (IoT) system verify the development of the proposed model. The prediction results show that the proposed predictor can obtain more accurate predictions of temperature, wind speed, and humidity data to meet the needs of precision agricultural production.

## 1. Introduction

Smart agriculture has been capable of offering many solutions to the modernization of agriculture [1]. With the development of Internet of Things (IoT) technology, smart agricultural applications have developed greatly [2,3,4] in recent years. Thanks to IoT systems, in which a wireless sensor network collects data from sensors deployed at various nodes and sends data over a wireless protocol, the massive data from the IoT agriculture system can be collected, such as temperature, wind speed, and humidity, which can provide information about environmental factors, enabling climate predictions.

The agricultural industry is susceptible to the climate, and a comprehensive understanding of future climate information can generate more benefits for smart agricultural development. The climate prediction has high reference value. Small climate stations in agricultural areas monitoring and predicting climate changes in real-time can help farmers quickly adjust a planting plan to minimize losses. For the prevention of, and resistance to, risks in the agricultural industry, the support of climate prediction technology is indispensable. The estimation and prediction of climate changes are often based on mathematical models. Some of the predicted models can be established through certain parameter estimation methods [5,6,7,8], some use input–output representations [9,10,11], while others use state–space models [12] or network models [13,14].

To apply the IoT technology to the development of modern agriculture, many researchers have made many outstanding contributions [15,16,17]. One example is an agricultural Internet of Things system that is used to help monitor plant production processes and enable early warning and rapid diagnosis of major pests and diseases [18]. Hao et al. [19] studied the relationship between farmland environmental factors and crop growth cycle and crop yield based on agricultural big data. Zou et al. [20] proposed a new service-based, grid-based approach to building agricultural IoT systems. Agricultural material information can be used in agricultural engineering and has obvious advantages.

**Figure 1:**
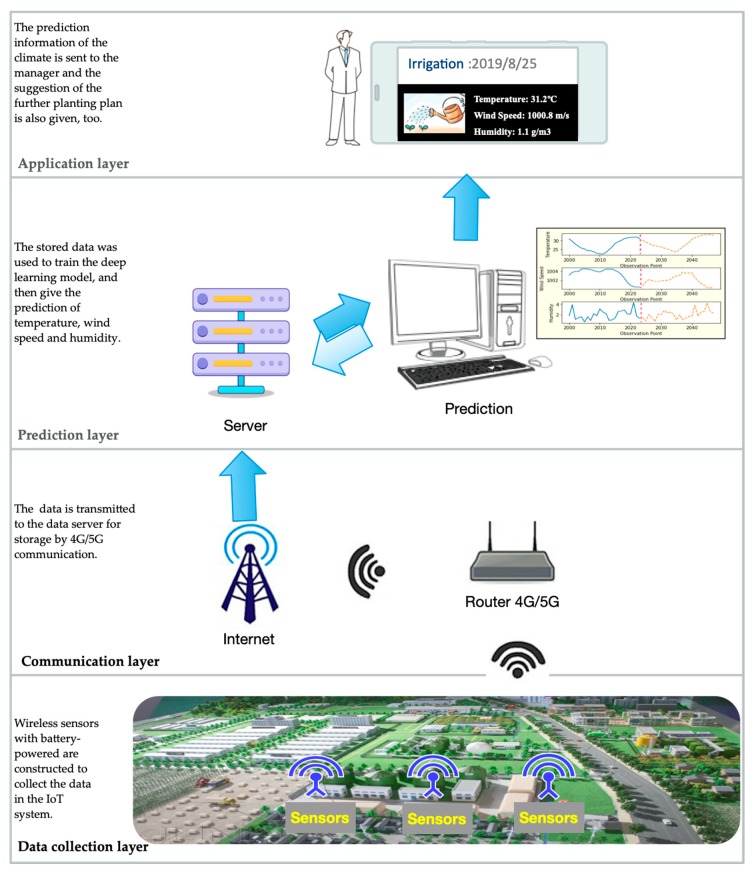
Data collection and prediction in an agricultural Internet of Things (IoT) system.

Figure 1 shows a schematic diagram of data collection, communication, prediction, and application layers in agricultural IoT systems. Because the planting is outdoor, battery-powered wireless sensors are constructed to collect the data in the IoT system. The sensors are used to measure the temperature, wind speed, and humidity, then the data is transmitted to the data server for storage by 4G/5G communication. Furthermore, a large quantity of stored data is used to train the deep learning model and to then give the predictions of future temperature, wind speed, and humidity. The climate prediction information is then sent to the manager, and a suggestion for a future planting plan is also given. 

The prediction and evaluation of climate trends can assure the quality of agricultural products; in addition, the improvement of climate prediction accuracy in agricultural production areas will help to better protect the development of agriculture. Prediction based on climate data is a difficult problem because data collected from sensors have complex nonlinear relationships with multiple components and are polluted by noise. Therefore, it is impossible to make accurate long-term predictions, but researchers are also convinced that accurate medium-term predictions are possible.

On the other hand, thanks to their high sensing frequency, large-scale data can be collected and stored by IoT systems, making it possible to analyze sensory data, discover new information, and gain insights [21] by using artificial intelligence methods such as deep learning.

This study focuses on medium-term prediction in an agricultural IoT system by processing the collected sensing data with artificial intelligence methods. Medium-term prediction means predicting 20 to 30 steps ahead. As for the climate data collected by the agricultural IoT system shown in Figure 1, we provide a prediction of the temperature, wind speed, and humidity 24 steps ahead based on the deep learning method. To overcome the highly complex nonlinearity, the climate data are first decomposed, and then the deep learning network is used to model component groups. The proposed method can accurately predict changes in the next 24 hours to meet the needs of precision agricultural production the next day.

The remainder of this study is organized as follows. Section 2 introduces the current prediction technology, discusses its advantages and disadvantages, and explains our innovations. Section 3 describes each part of the proposed predictor. Experimental results of the proposed predictor are shown and evaluated in Section 4, and finally Section 5 presents our conclusion.

## 2. Related Work

As a key data processing technology, prediction methods have been widely used in science, engineering, and other fields. However, since the dynamics of collected sensing data are usually strongly nonlinear, the prediction methods still face enormous challenges. 

Time series prediction methods can be roughly divided into two categories: single methods and combined methods. We discuss these 2 kinds of prediction methods in the following subsection, and then based on the current research results, we propose our innovative method.

### 2.1. Single Methods 

Traditional methods include physical analysis models and prediction models based on statistical theory. In the physical models, the equations are established based on the physical relationships between variables, and then the prediction models analyze and predict time series data [22]. Obviously, a suitable mathematical model should be built to fit the historical curve, and the future trend is predicted according to the model trend curve. Statistical models used include the autoregressive moving average (ARMA) [23,24,25], autoregressive integrated moving average (ARIMA) [26], threshold autoregressive (TAR) model [27], and the hidden Markov model (HMM) [28]. For example, Kumarmahto et al. [29] selected the ARIMA (1,1,2) model for the prediction of agriculture commodity prices.

Based on theoretical assumptions and prior knowledge, model parameters have to be set for these traditional methods. However, due to a lack of background knowledge, this is often difficult. Therefore, traditional methods are less commonly used in complex agricultural applications. Unlike traditional prediction methods, machine learning methods do not require prior physical information, meaning prediction models can be built based on learning algorithms and historical data. Prediction is obtained based on mathematical models, and some parameter identification methods can be used, such as iterative algorithms [30,31,32], particle-based algorithms [33,34], and recursive and learning algorithms [35,36,37,38,39].

Regression models were initially used for prediction tasks. Bhuriya et al. [40] applied linear regression methods to predict stock prices. Oteros et al. [41] established a multiple regression model by using different factors of pollen concentration to take into account extreme climate events in the Mediterranean climate. Donnelly et al. [42] proposed a method for predicting air quality based on multiple linear regression. These linear regression models face challenges in prediction tasks with highly nonlinear time series data.

Artificial neural networks (ANNs) play a key role in solving nonlinear problems. Mao et al. [43] developed a short-term wind prediction system based on a back-propagation (BP) neural network for numerical climate prediction data, such as wind speed, wind direction, temperature, relative humidity, and atmospheric pressure data. Theoretically, the BP neural network has been proven to enable fit any persistent nonlinear function, however problems exist, such as falling into local minimum values, over-fitting, and excessive training times. These problems can be corrected by optimization. For example, in [44], the particle swarm optimization (PSO) algorithm was combined with the BP network for short-term load prediction of power grids. The study showed that the prediction performance of the proposed model based on the PSO algorithm was better than that of a traditional BP neural network. Bai et al. studied the combined prediction method of a shallow nonlinear autoregressive network (NAR) on the basis of BP [45] and proposed the prediction method from time and space dimensions by using shallow networks [46].

Later studies compared the optimized results with the results of regression models (e.g., [47,48]), proving the effectiveness of the optimization algorithm and promoting the combination of optimization algorithms and neural networks as predictive tools. A recurrent neural network (RNN) [49] for time series prediction has attracted extensive attention from researchers because it could capture the high nonlinearity of time series data. Yadav et al. [50] used climate data to predict average solar radiation through RNN and proposed an adaptive learning rate for RNN. As an improved version of RNN, long short-term memory (LSTM) replaced it and became a popular time series data prediction technology [51,52]. A gated recurrent unit (GRU) [53] inherits the advantages of LSTM, can automatically learn features and model long-term dependent information, and also shows an improvement in calculation speed.

This intelligent method has been applied to intelligent agriculture. Alreshidi [54] explored artificial intelligence technologies for smart, sustainable agriculture. Pantazi et al. [55] used machine learning to predict wheat yield based on online multilayer soil data and satellite imagery crop growth characteristics, and showed that the supervised Kohonen networks had the best overall performance. To utilize scalability for yield prediction, Oliveira et al. [56] developed the geographically weighted gradient boosting machine (GW-GBM), which was essential to agriculture stakeholders. In [57], authors studied comparisons of deep learning with other existing popular techniques with respect to differences in classification or regression performance. The findings indicated that deep learning provided high accuracy, outperforming commonly used image processing techniques.

Deep learning is used to extract accurate sensor data information from IoT devices deployed in complex environments [58]. In [59], the performance of IoT deep learning applications was optimized by edge computing. As a review, [21] provided a thorough overview of the use of a deep learning method to facilitate analysis and learning in the IoT domain, and pointed out that deep learning models produce high-level abstraction and insight that is fed into the IoT systems to fine-tune and improve services. Fast data analysis and medium- or long-term prediction of collected sensing data are still challenging research aspects.

### 2.2. Combined Methods

In recent years, combined methods based on machine learning have been proven effective at improving prediction performance, and various hybrid models have been introduced to predict nonlinear time series data. Differing from the so-called single methods in Section 2.1, a combined method here means that several methods are used together in a parallel or serial structure. For example, Yahata et al. [60] combined machine learning techniques to construct sensing methods in an agricultural cyber–physical system, in which big data of agricultural plants and environmental information (e.g., temperature, humidity, solar radiation, soil condition, etc.) were analyzed to mine useful rules for appropriate cultivation. Wu et al. [61] proposed a mixed model, which combined the ARIMA model and regression method based on time and space factors, and produced warnings for daily price changes using neural networks. 

A seasonal trend decomposition procedure based on loess, the sequential two-level method, was used to model pollen time series in the air, and this was then used to predict the daily pollen concentration for the next period [62]. The authors explained that analyzing each component of the data separately can identify the source of change in data more accurately than the original undecomposed series. Xiong et al. [63] proposed a novel hybrid method combining STL and extreme learning machines (ELMs) to predict seasonal vegetable prices, which contributed to the development of agriculture. Liu et al. [64] implemented a hybrid method to predict wind speed, in which the wavelet transform was used to decompose the wind speed signal into two components and the approximated signal (one of two components) was modeled by a support vector machine. Zhi et al. [65] selected empirical mode decomposition (EMD) as the decomposition method for the time series, and components with different features in the original hydrological time series were decomposed. Yaslan et al. [66] predicted power load demand using a combined EMD and support vector regression (SVR) model. The decomposition process of EMD was regarded as a denoising procedure for the training data, and the prediction results were compared with those of the SVR algorithm based on different feature vectors. The results showed the superiority of the model in power load prediction.

Through EMD, the mode function (IMF) component is a local characteristic signal. The IMF is based on different time scales of the original time series itself, representing each frequency component in the original signal, which are arranged independently from each other in order from high to low. The EMD decomposition process is a simplification of complex time series. For example, the first high-frequency IMF sequence is treated as a noise term and discarded [67,68].

Qiu et al. [69] proposed a hybrid method based on EMD and deep learning, in which the load demand sequence is decomposed into IMF. The extracted IMF is then modeled using a deep belief network (DBN) to accurately predict the evolution of each IMF. Finally, the predictions of each model are combined by addition to obtain the total output of the load demand. Wang et al. [70] introduced a feedforward neural network (FNN) into an EMD-based prediction framework, proposed a weighted recombination strategy, and performed single-step prediction experiments on four nonlinear, nonstationary sequences. Bedi et al. [71] combined EMD with LSTM to estimate the power demand over a given time interval. The performance of this method was evaluated by comparing it the prediction results of the EMD of RNN and RMD models. EMD can result in different numbers of trained and online predicted models, but [69,70,71] does not explain how to solve this problem. Significantly differing from previous studies, we will combine IMF components to achieve the unification of the training model and prediction model in practical applications.

We continue the combination of EMD together with deep learning networks, and our innovative contributions are highlighted as follows.

(1) The obtained IMF components are analyzed with frequency characteristics, and all the components are divided into 3 groups using a learning network based on convolution operation. Differing from [69,70,71], this method can solve the “different number problem” of IMF components.
(2) We present a general agricultural IoT system framework for predicting climate data and obtain accurate medium-term predictions that can meet the needs of precision agricultural production.

## 3. Hybrid Deep Predictor

The proposed predictor has a hybrid structure, for example using EMD decomposition to reduce the nonlinear complexity and dividing IMFs into 3 groups using Convolution Neural Networks (CNN) networks. For each group, the deep learning network GRU is used for modeling and prediction, and finally all the predictions of the GRU are added to obtain the prediction result.

### 3.1. Decomposition and Analysis for Time Series

The EMD method decomposes complex signals into a finite number of IMFs based on the frequency characteristics, which should satisfy the following conditions: (1) the absolute value of the difference between the number of zero crossings and extreme points is equal to 0 or 1; and (2) the mean value of the envelope constructed by local maxima and minima must be zero at any point. EMD is an adaptive data processing or mining method and is essentially a smoothing process for time series data.

Assume $D_{t}$ is the time series to be decomposed and $h_{e}$ is the expected decomposition result to be obtained. The decomposition process is as follows [72]:

(1) Fit the maximum and minimum points of $D_{t}$ with the cubic spline interpolation function to form the upper and lower envelope.
(2) Calculate the mean of the upper envelope and the lower envelope, denoted as $m_{e}$.
(3) Subtract the mean of $m_{e}$ by $D_{t}$ to obtain a new data sequence $h_{e}$: $h_{e}=D_{t}-m_{e}$.
(4) Repeat steps 1-4 until one of the following stop criteria is met: (1) the preset maximum number of iterations is reached; (2) the last separated IMF is small; (3) the maximum or minimum value of the signal is less than 2; (4) $h_{e}$ is a monotonic curve.
(5) Treat $h_{e}$ as an IMF, and calculate the remainder $R_{t}=D_{t}-h_{e}$.
(6) Use $R_{t}$ as the new $D_{t}$, and repeat steps (1)–(6) until all IMFs are obtained.

**Figure 2:**
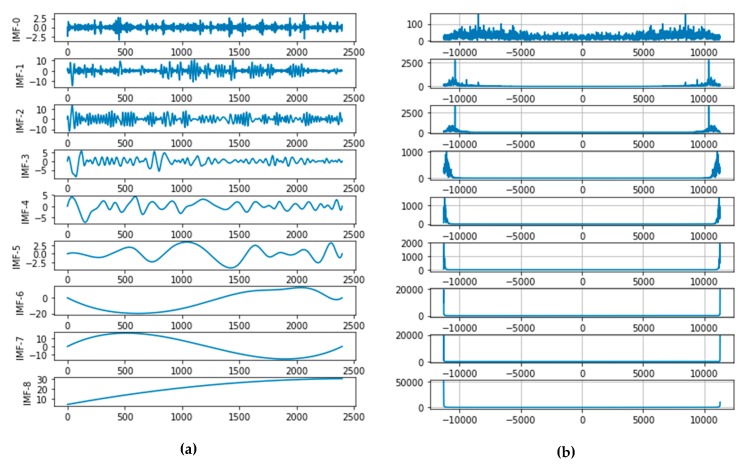
Correspondence of each mode function (IMF) between time and frequency domains after decomposition. IMFs in the (**a**) time domain and (**b**) frequency domain.

We take temperature data as an example to give the results of EMD decomposition. In Figure 2a, all the obtained IMFs are shown over time (from IMF-0 to IMF-8), and correspondingly each sub-picture on the right-hand side (b) is the frequency component. It can be found that each IMF has a specific time and frequency domain, and the frequency components contained in the IMF are reduced from top to bottom.

**Figure 3:**
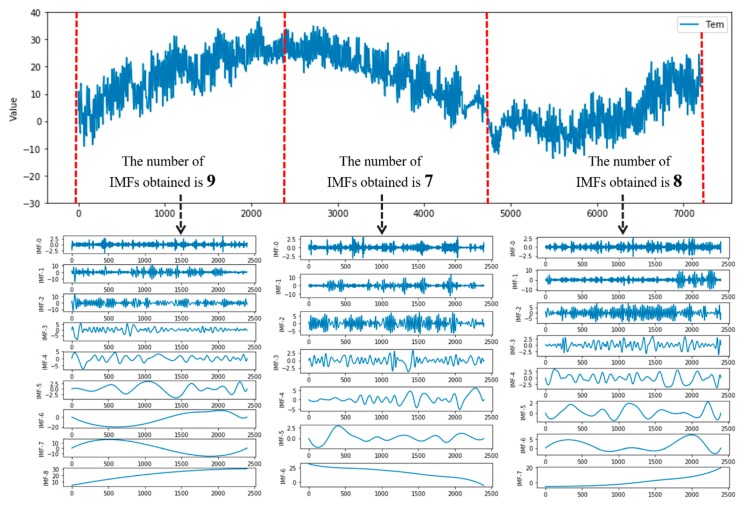
The number of IMFs within different time intervals of temperature data.

Moreover, we found that the number of IMFs varies for different periods. As shown in Figure 3, we performed EMD on the three different data intervals [0, 2400), [2400, 4800), and [4800, 7200] of the temperature data, and the number of IMFs obtained was 9, 7, and 8, respectively.

We can note that the number of trained prediction sub-models will be different from the number of IMF components in the training prediction interval. Therefore, it is necessary to combine IMFs into a fixed number according to frequency characteristics.

In this study, according to the frequency characteristics of IMFs, we combined all the IMFs into 3 groups, meaning the decomposition components with similar frequency characteristics will be labeled, grouped, and added together, then for each group, one model will be trained. Therefore, the number of models in each prediction interval will be fixed.

### 3.2. Classification and Combination for IMFs

**Figure 4:**
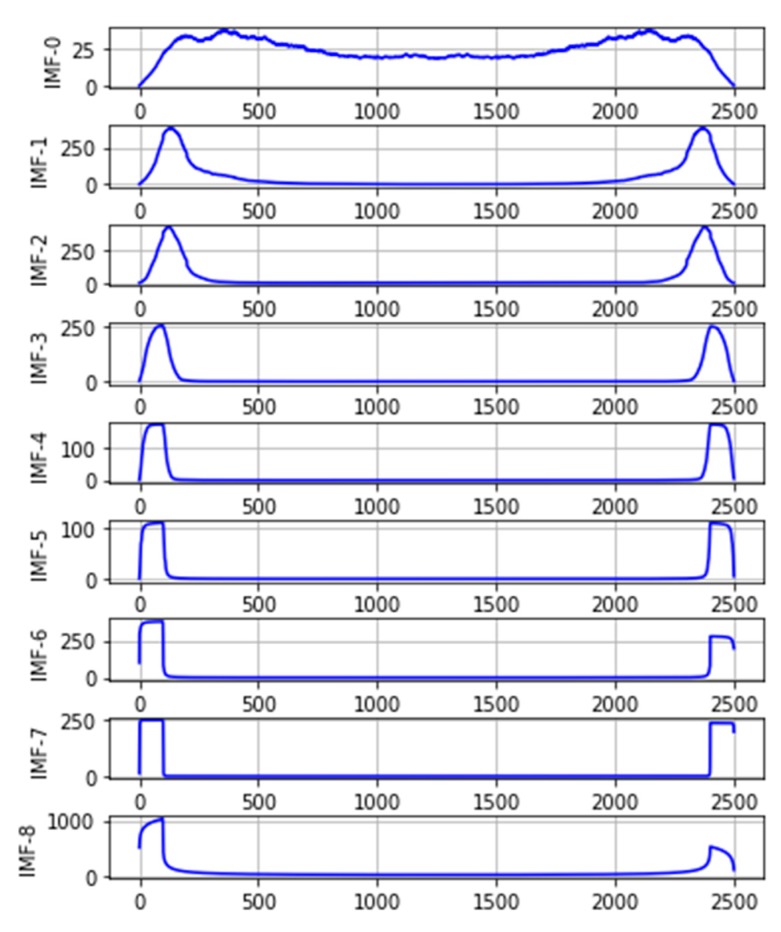
Convolution results for each IMF in the frequency domain.

Figure 4 shows that a one-dimensional convolution operation is calculated for each IMF in the frequency domain to illustrate the different frequency components, with the convolution formula as follows:

(1) $$f(x)*g(x)=\int_{-\infty }^{+\infty }f(\tau )*g(x-\tau )d\tau$$

where f(x) is the convolved function and g(x) is the convolution kernel function. The result of the one-dimensional convolution is equal to the integral of the integrand function f(τ)*g(x−τ) on the interval (−∞,+∞) and the convolution kernel g(x) is selected as a Gaussian kernel function.

The results in Figure 4 show that IMF-0 contains a wider frequency band. In contrast, IMF-1 and IMF-2 are significantly reduced, but there are still long tails in the cutoff band. Differing from IMF-3 and IMF-4, the downhill is significantly steeper, indicating that fewer frequency components are included. Furthermore, for IMF-5–8, the downslope is almost vertical, and we find that the fluctuations of these components on the time domain map (Figure 2a) are relatively flat.

Figure 4 has shown that the convolution operation can capture the dynamic change of data, so we use the CNN neural network to classify and group IMFs. A one-dimensional CNN is used for feature extraction from IMF sequences. Given an input IMF sequence $X_{t}$, t=1,…,n and the convolution kernel function, the filters sequentially perform a local convolution operation on the input features of the previous layer. The output of the convolution is as follows:

(2) $$x_{t}=\sum_{l=1}^{m}k_{l}\times X_{t-l+1}.$$

The rectified linear unit (ReLU) with fast convergence speed is selected as the activation function as follows:

(3) $$f(x_{t})=\{\begin{array}{ll} 0, & x_{t}\leq 0\\ x_{t}, & x_{t}>0 \end{array}$$

**Figure 5:**
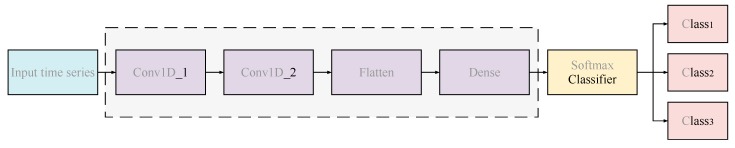
Schematic of a one-dimensional convolutional neural network (CNN).

Then using flattening and full connection processes [73], a one-dimensional CNN is used to extract the frequency characteristics of the IMFs, using the Softmax classifier to classify the features, finally achieving the network output (i.e., the labels for each IMF). The schematic of the one-dimensional CNN is shown in Figure 5.

### 3.3. Deep Prediction Network for Combined IMFs

**Figure 6:**
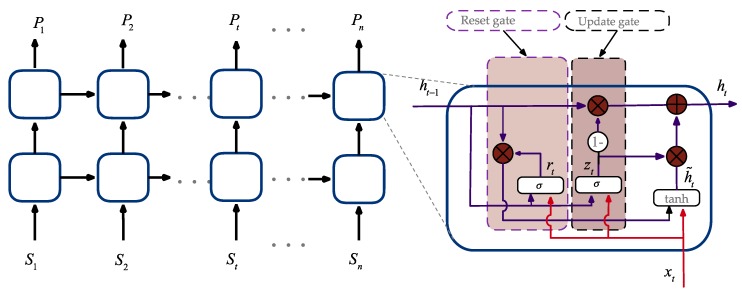
The network structure of the gated recurrent unit (GRU).

Using the known input and output data, the GRU is trained by the stochastic gradient descent algorithm and the optimal weight can be obtained. The GRU network was trained on the sum of IMF sequences in each group. The GRU network consisted of multiple GRU cells, and here the number of hidden layers is set as 2. Shown as Figure 6, $S_{t}$, t=1,2,…,n is the input of the GRU network and $P_{t}$, t=1,2,…,n is the output.

The GRU cell uses the update gate to control the degree to which the state information of the previous moment is brought into the current state. The larger the value of the update gate, the more the state information is brought in at the previous moment. The reset gate is similar to the forget gate of LSTM, which is used to control the degree to which the state information of the previous moment is ignored. The smaller the reset gate value, the more information is neglected.

The forward propagation formulas in each GRU cell are as follows [74]:

(4) $$\begin{array}{l} z_{t}=\sigma (a_{t}U^{z}+h_{t-1}W^{z}+b^{z})\\ r_{t}=\sigma (a_{t}U^{r}+h_{t-1}W^{r}+b^{r})\\ {\tilde{h}}_{t}=\tanh(a_{t}U^{h}+(h_{t-1}\circ r_{t})W^{h}+b^{h})\\ h_{t}=(1-z_{t})\circ {\tilde{h}}_{t}+z_{t}\circ h_{t-1} \end{array}$$

where $a_{t}\in R^{d}$ is the input vector of each GRU cell; $z_{t}$, $r_{t}$, ${\tilde{h}}_{t}$, and $h_{t}$ stand for the update gate, reset gate, candidate state of the current hidden node, and the active state of the current hidden node output at time t, respectively; U and W are weight matrices to be learned during model training; b represents bias vectors; ∘ is an element-wise multiplication; and σ and tanh are activation functions. The GRU is trained by the gradient descent algorithm and the parameters are continually updated until convergence. The methods proposed in this paper can be applied to other fields, such as water environment prediction and management control systems [75], IoT intelligent systems [76,77,78], and wireless sensor networks [79,80,81].

### 3.4. Model Framework for Smart Agriculture Sensing

**Figure 7:**
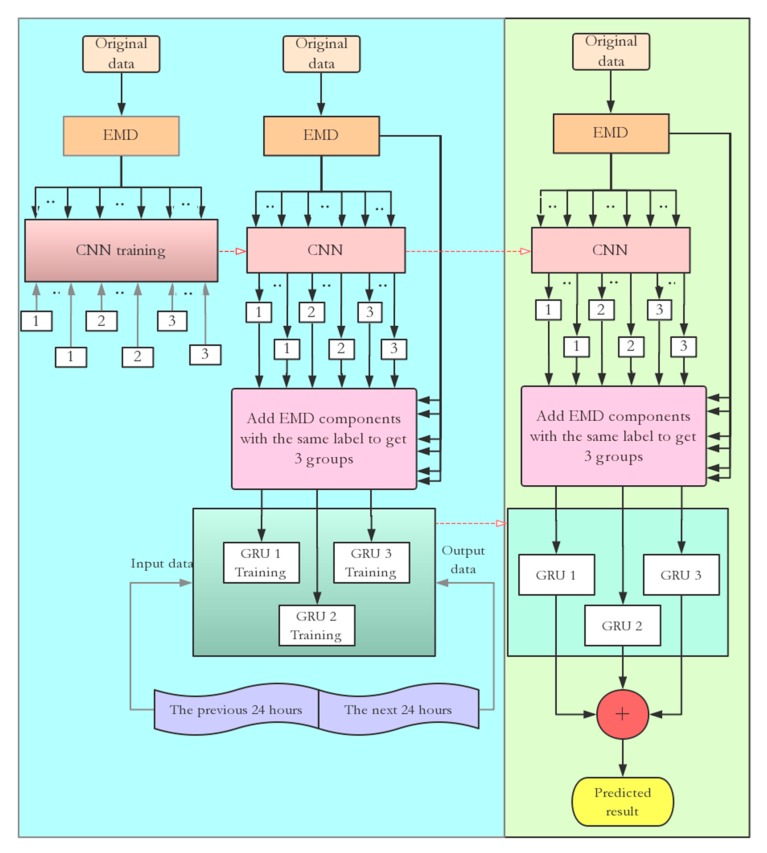
Flowchart of a hybrid predictor for smart agriculture sensing.

In conclusion, based on the discussion in Section 3.1, Section 3.2 and Section 3.3, the proposed deep learning predictor is shown in Figure 7. The number of groups is fixed to 3. The model includes the two processes of training and prediction. The first process trains the CNN and GRU based on the IMFs decomposed by EMD. The data is decomposed into IMFs by EMD and the labels are set for each IMF, which are then separated into 3 groups based on frequency characteristics and assigned as Groups 1-3. Then, the CNN is trained by IMFs and labels, and the sequences are added to each group. Finally, GRU models are trained for each group to obtain three GRU sub-predictors.

The prediction process predicts the future trends of climate data using the trained networks, and it is implemented by summing all the group predictions of GRU models based on IMF groups. The details are given in Figure 7.

## 4. Experiment Results and Discussion

### 4.1. Dataset and Experimental Setup

Our experiments are based on the collected sensing data of an agricultural IoT system in Beijing, which collected hourly climate data, including temperature, wind speed, and humidity. The data was obtained from 2016 to 2018 and consisted of 20,013 time series data points. This dataset will be used to train and test the proposed model and other models in Section 4.2 and Section 4.3. In the experiments, the ratio of training to testing sets was 7:3. We need to predict temperature, wind speed, and humidity 24 h ahead of time.

The open-source deep learning library Keras, based on TensorFlow, was used to build all the learning models. All the experiments were performed on a PC server with an Intel CORE CPU i5-4200U at 1.60 GHz, with 4 GB of memory.

To effectively model the deep neural network, hyper-parameters were set based on experience from experiments. The default parameters in Keras were used for deep neural network initialization (e.g., weight initialization). The activation function of the CNN models is ReLU. The CNN had 2 convolutional layers, with 32 convolution kernels in each layer and a logistic loss function. The kernel size was set as 5. The input and output lengths of the CNN were 24. The identification labels were set by one-hot encoding.

The GRU was designed with two layers and the number of neurons was set at 24. For GRU, the Adam method is used by optimizing a predetermined objective function and Huber loss is used to obtain the robust prediction result, because the sensing data always contain noise from the IoT system. The subsequences were trained using GRU to establish sub-predictive models. Tanh is used as the activation function of GRU. In the experiments, the prediction was considered 24 steps ahead, in which we used the climate data during the previous 24 h to predict the next 24 h.

In Case 1 and Case 2, the root mean square error (RMSE) (shown in Equation (5)) was used to measure the difference between the prediction and the collected data.

(5) $$RMSE=\sqrt{\frac{{\sum_{i=1}^{N}\left(x_{pre}(i)-x_{obs}(i)\right)}^{2}}{N}}$$

where N is the number of the predictive datasets; $x_{obs}$ represents the collected data and $x_{pre}$ is predicted value.

### 4.2. Case 1: Prediction Performance Analysis of Different Predictors

In this experiment, 6 models are used for comparison with the proposed method, which are RNN [49], LSTM [51], GRU [53], EMDCNN_RNN [49] and EMDCNN_LSTM [51] (which are obtained by decomposing the data using EMD and with classification of the CNN) as the sub-predictors, and finally the sequential two-level method (STL) method from [17]. The temperature, wind speed, and humidity data introduced in Section 4.1 are used to show the prediction result.

In Table 1, a comparison between the proposed method and RNN [49], LSTM [51], GRU [53], EMDCNN_RNN [49] (EMD and CNN-based RNN [49]), EMDCNN_LSTM [51] (EMD and CNN-based LSTM [51]), and STL [17] in terms of RMSE.

**Figure 8:**
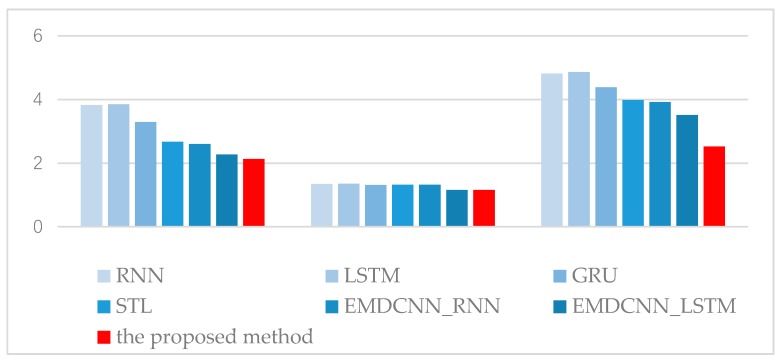
Histogram of RMSE values for predictions of temperature, wind speed, and humidity.

**Figure 9:**
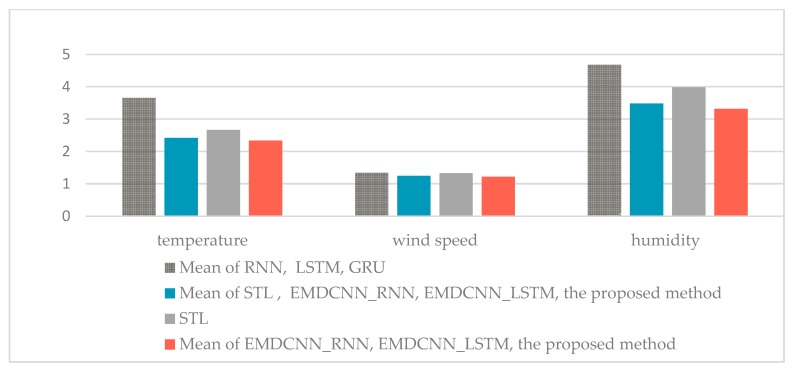
The histogram of different RMSE values.

Table 2 gives comparisons the means between the proposed method and RNN [49], LSTM [51], and GRU [53]; between the proposed method and STL, EMDCNN_RNN, and EMDCNN_LSTM; and between the proposed method and EMDCNN_RNN and EMDCNN_LSTM. Figure 8 and Figure 9 give the histogram of RMSEs in Table 1 and Table 2.

The results show that firstly, the decomposition is necessary as the RMSEs can be significantly reduced. We obtained the mean RMSEs of the decomposition methods of EMD and STL as 2.4165, 1.24055, and 3.48356 for temperature, wind speed, and humidity, respectively. These values are much less than those obtained for single models for RNN, LSTM, and GRU, which were 3.6551, 1.3375, and 4.6855, respectively.

Moreover, EMD outperforms the STL method. The mean RMSEs of all the EMD methods were 2.333, 1.2127, and 3.3177, respectively, which were less than the results of STL. Further, we find that GRU is the best choice as the sub-predictor in Table 1. The prediction RMSE values of the proposed method for temperature were approximately 18.01% and 6.07% lower, respectively, than those of EMDCNN_RNN and EMDCNN_LSTM; for wind speed, the RMSEs were about 12.95% and 0.57% lower, respectively; and for humidity, the RMSEs were about 35.77% and 28.29% lower, respectively. This shows that the proposed method, which used GRU as the sub-predictor, gave the best performance. These improvements are beneficial to agricultural IoT systems. The development of the proposed method means that the temperature prediction accuracy is increased by about 1 degree, which is important for agricultural production.

### 4.3. Case 2: Prediction Performance Analysis of Different Combinations for IMFs

In this experiment, the collected hourly climate data, including temperature, wind speed, and humidity, in Beijing from January 2016 to January 2018 are used to show the prediction result. We conclude that mode no. 5 (which includes 3 groups, namely Group1: {IMF 0-2}, Group2: {IMF 3-4}, and Group3: {IMF 5-8}) is the suitable mode for the proposed system.

**Figure 10:**
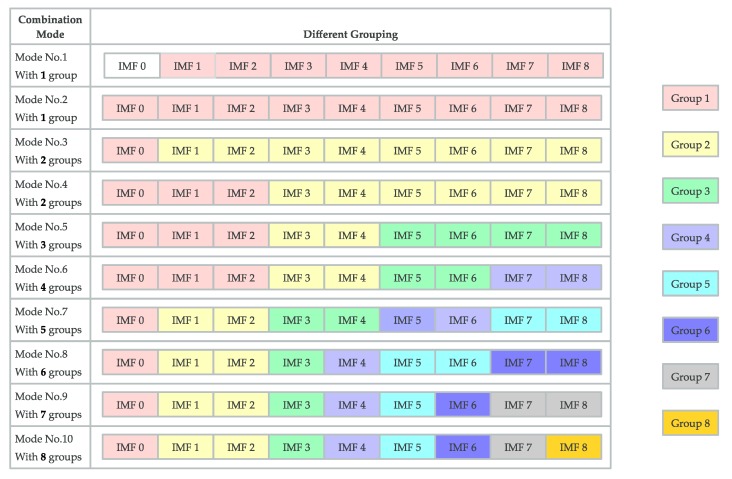
Different modes with different groups shown by different color blocks.

The 10 different modes with different groups are shown in Figure 10 using different color blocks. For example, in mode no. 1, IMF0 is discarded, and the other IMFs are added together as Group 1. As for mode no. 2, all the IMFs are added together as Group 1. Further, mode no. 3 has two groups (i.e., Group 1 (with IMF 0) and Group 2 (the sum of IMF 1 to IMF 8)) and we use 2 color blocks in the third line of Figure 9. Similarly, mode no. 10 has 8 groups, namely Group 1 (with IMF 0), Group 2 (with the sum of IMF 1 and IMF 2), Group 3 (with IMF 3), Group 4 (with IMF 4), Group 5 (with IMF 5), Group 6 (with IMF 6), Group 7 (with IMF 7), and Group 8 (with IMF 8).

**Figure 11:**
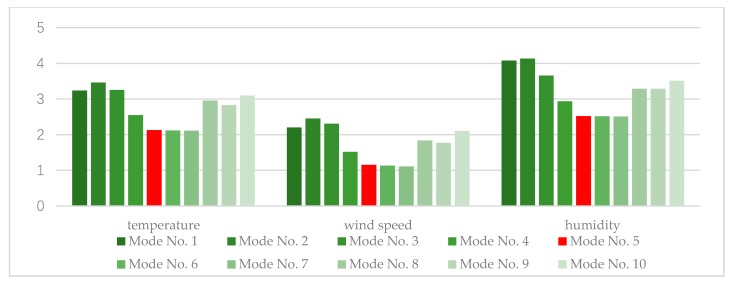
Histogram of numerical comparisons of RMSEs for different combination modes.

For each mode, we used the different combination modes to train the CNN and obtained different numbers of groups. Then, GRUs were trained for each group. Table 3 gives the comparison of RMSEs of prediction results with different groupings, and Figure 11 shows the histogram of numerical comparisons of RMSEs.

The data in Table 3 and Figure 11 can be interpreted as a large difference in performance across the different groups. For the temperature data, the RMSE of {IMF 1-8} in mode no. 1 is 6.56% lower than mode no. 2, which indicates that removing the first component (noise item) decomposed by EMD is helpful for prediction. The RMSE decreases from mode no. 2 to mode no. 7, but prediction increases in mode no. 8 and mode no. 10. We can note that the performance will be worse if the number of groups exceeds 5.

Mode no. 5, mode no. 6, and mode no. 7 have similar prediction performance, with RMSE reaching 2.1310, 2.1556, and 2.1093, respectively. Compared with mode no. 5, the RMSE of mode no. 6 or mode no. 7 is slightly reduced, but we have to train 4 or 5 GURs, so the training parameters are increased by one-third and two-thirds, respectively. In addition to the accuracy of the predictions, we believe that the amount of computation of the IoT system is also important. To reduce the cost of training, a method that requires less calculation should be selected. Therefore, to ensure prediction performance and maintain the cost of parameters, mode no. 5 with 3 groups is a good choice for application of temperature data in agricultural IoT systems.

For wind speed and humidity data, we obtained similar results, so in this study 3 group modes are used for the proposed predictor.

## 5. Conclusions

In recent years, predictions based on collected sensing data have become an important aspect of IoT applications in many fields, and deep learning has been the most widely used method in many IoT systems because it has excellent nonlinear modeling capabilities.

Through the establishment of climate monitoring stations in agricultural areas, various climate data are collected. In actual IoT agricultural systems, IoT technology has been widely used to predict key climate factors in real time. A three-dimensional network of agro-meteorological data has been built, which has greatly promoted the development of agriculture.

This study proposes a hybrid deep learning predictor based on a learning EMD and GRU group model. The proposed method decomposes data by EMD and extracts local feature components using CNN, then uses a learning network based on convolution operation to classify IMFs based on the frequency feature and trains the GRU as the sub-predictor. The prediction results of the sub-predictor are finally added to obtain the final prediction result.

The proposed predictor has been used to predict temperature, wind speed, and humidity data in an agricultural IoT system. In practical applications, the proposed predictor can obtain accurate predictions for the following 24 hours, providing sufficient climate information for precision production.

## Figures and Tables

**Table 1:** Comparison of root mean square error (RMSE) of prediction results with the different predictors.

Element	RMSE						
Data	recurrent neural network(RNN) [40]	long short-term memory(LSTM) [42]	gated recurrent unit(GRU) [56]	sequential two-level method (STL) [17]	EMD and CNN-based RNN(EMDCNN_RNN)	EMD and CNN-based LSTM(EMDCNN_LSTM)	The Proposed Method
Temperature	3.8273	3.8442	3.2939	2.6672	2.5992	2.2688	**2.1310**
Wind speed	1.3472	1.3499	1.3154	1.3241	1.3249	1.1599	**1.1533**
Humidity	4.8143	4.8578	4.3844	3.9811	3.9215	3.5128	**2.5189**

**Table 2:** Comparisons of the means of RMSE.

Element	RMSE			
Data	Mean of RNN [40], LSTM [42], and GRU [56]	Mean of STL [17], EMDCNN_RNN, EMDCNN_LSTM, and the proposed method	STL [17]	Mean of EMDCNN_RNN, EMDCNN_LSTM, and the proposed method
Temperature	3.6551	2.4165	2.6672	2.333
Wind speed	1.3375	1.2405	1.3241	1.2127
Humidity	4.6855	3.4836	3.9811	3.3177

**Table 3:** Comparison of RMSEs of prediction results with different groupings.

Combination Mode	Number of Groups	RMSE		
		Temperature	Wind Speed	Humidity
Mode No. 1	1 group	3.2354	2.1989	4.0798
Mode No. 2	1 group	3.4626	2.4560	4.1343
Mode No. 3	2 groups	3.2558	2.3054	3.6562
Mode No. 4	2 groups	2.5474	1.5152	2.9345
Mode No. 5	3 groups	**2.1310**	**1.1533**	**2.5189**
Mode No. 6	4 groups	2.1156	1.1321	2.5166
Mode No. 7	5 groups	2.1093	1.1102	2.5101
Mode No. 8	6 groups	2.9550	1.8350	3.2859
Mode No. 9	7 groups	2.8293	1.7685	3.2855
Mode No. 10	8 groups	3.0985	2.1026	3.5113
